# Observational Dutch Young Symptomatic StrokE studY (ODYSSEY): study rationale and protocol of a multicentre prospective cohort study

**DOI:** 10.1186/1471-2377-14-55

**Published:** 2014-03-22

**Authors:** Renate M Arntz, Mayte E van Alebeek, Nathalie E Synhaeve, Paul J Brouwers, Gert W van Dijk, Rob A Gons, Tom den Heijer, Paul LM de Kort, Karlijn F de Laat, Anouk G van Norden, Sarah E Vermeer, Maureen J van der Vlugt, Roy PC Kessels, Ewoud J van Dijk, Frank-Erik de Leeuw

**Affiliations:** 1 Radboud University Medical Center, Donders Institute for Brain, Cognition and Behaviour, Center for Neuroscience, department of Neurology, PO Box 9101, 6500, HB Nijmegen, the Netherlands; 2 Department of Neurology, St. Elisabeth Hospital, PO Box 90151, 5000, LC Tilburg, the Netherlands; 3 Department of Neurology, TweeSteden Hospital, PO Box 90107, 5000, LA Tilburg, the Netherlands; 4 Department of Neurology, Medisch Spectrum Twente, PO Box 50000, 7500, KA Enschede, the Netherlands; 5 Department of Neurology, Canisius-Wilhelmina Hospital, PO Box 9015, 6500, GS Nijmegen, the Netherlands; 6 Department of Neurology, Catharina Hospital, PO Box 1350, 5602, ZA Eindhoven, the Netherlands; 7 Department of Neurology, Sint Franciscus Gasthuis, PO Box 109000, 3004, BA Rotterdam, the Netherlands; 8 Department of Neurology, Haga Hospital, PO Box 40551, 2504, LN Den Haag, Netherlands; 9 Department of Neurology, Amphia Hospital, PO Box 90157, 4800, RL Breda, the Netherlands; 10 Department of Neurology, Rijnstate Hospital, PO Box 9555, 6800, TA Arnhem, the Netherlands; 11 Radboud University Medical Centre, department of Cardiology, PO Box 9101, 6500, HB Nijmegen, the Netherlands; 12 Department of Medical Psychology, Radboud University Medical Centre, PO Box 9101, 6500, HB Nijmegen, the Netherlands; 13 Donders Institute for Brain, Radboud University Nijmegen, Cognition and Behaviour, Nijmegen, the Netherlands

**Keywords:** Young adults, Stroke, TIA, Prognosis, Risk factors

## Abstract

**Background:**

The proportion of strokes occurring in younger adults has been rising over the past decade. Due to the far longer life expectancy in the young, stroke in this group has an even larger socio-economic impact. However, information on etiology and prognosis remains scarce.

**Methods/design:**

ODYSSEY is a multicentre prospective cohort study on the prognosis and risk factors of patients with a first-ever TIA, ischemic stroke or intracerebral hemorrhage aged 18 to 49 years. Our aim is to include 1500 patients. Primary outcome will be all cause mortality and risk of recurrent vascular events. Secondary outcome will be the risk of post-stroke epilepsy and cognitive impairment. Patients will complete structured questionnaires on outcome measures and risk factors. Both well-documented and less well-documented risk factors and potentially acute trigger factors will be investigated. Patients will be followed every 6 months for at least 3 years. In addition, an extensive neuropsychological assessment will be administered both at baseline and 1 year after the stroke/TIA. Furthermore we will include 250 stroke-free controls, who will complete baseline assessment and one neuropsychological assessment.

**Discussion:**

ODYSSEY is designed to prospectively determine prognosis after a young stroke and get more insight into etiology of patients with a TIA, ischemic stroke and intracerebral hemorrhage in patients aged 18 to 49 years old in a large sample size.

## Background

Recent studies have shown that the proportion of strokes occurring in adults younger than 50 years of age has increased from 13 to 19% over the past decade [1,2]. Due to the longer life expectancy in the young compared with a general elderly stroke patient, stroke in this younger group has a large impact on number of years lost to ill-health, disability or early death [3]. This impact will not only be determined by the direct mortality and residual post-stroke deficit, but may also be influenced by future vascular events, cognitive impairment or post-stroke epilepsy that may occur throughout their post-stroke lives [4,5]. Especially for young patients reliable information on this prognosis is of great importance as their stroke occurs in the period of life in which they would like to form families, have an active social life and make decisive career moves. However, it is exactly the prognosis of this younger group that remains unclear.

Previous studies on stroke in the young were mainly retrospective and included patients who had their index events at times with a completely different secondary prevention strategy. Most studies only aimed at prognosis in terms of mortality and vascular events, which are very important outcome measures. However, other important post-stroke complications, such as cognitive deficits, post-stroke epilepsy and post-stroke functioning were not taken into account. Furthermore previous studies have only included patients with an ischemic stroke, whereas studies in younger patients with a transient ischemic attack (TIA) or intracerebral hemorrhage are scarce. There is growing evidence that despite the fact that TIA patients suffer from (by definition) transient neurological deficits, their cognitive or social sequelae may last way longer [6-8]. In addition, survival of patients with an intracerebral hemorrhage has improved substantially due to improved care resulting in longer post-stroke life expectancy. Information on prognosis after an intracerebral hemorrhage or TIA is consequently of great importance as well, both for patients and their health professionals when counselling their patients with information on the course of the disease.

Apart from uncertainties about prognosis and the major socio-economic impact of a young stroke, the explanation for the increased incidence of young stroke remains unclear. It has been suggested that an increased prevalence in traditional vascular risk factors, such as diabetes mellitus and obesity due to unhealthy life style and poor education, results in (more) atherosclerosis already at younger ages which may lead to stroke [9,10]. Furthermore, a rising incidence of substance abuse which may be more pronounced in the young might play a role [11]. However, despite the use of ever increasing additional clinical investigations that take place in renowned specialized stroke centers, etiology remains unknown in more than 30% of the young stroke patients [12]. An approach to get more insight into pathophysiological mechanisms of a young stroke, is to identify potentially acute trigger factors that precede a young stroke. In studies on subarachnoid hemorrhage and ischemic stroke in elderly, several acute trigger factors already have been identified (including for example vigorous physical exercise, emotions and coffee consumption) [13,14].

The assessment of prognosis and etiology is a first step in informing young stroke patients in terms of prognosis. We therefore will perform the Observational Dutch Young Symptomatic StrokE studY (ODYSSEY); a large multicentre prospective cohort study on prognosis and both traditional and other risk factors of patients with a TIA, ischemic stroke or intracerebral hemorrhage aged 18 through 49 years.

## Methods/design

### Study design

ODYSSEY is a multicentre prospective cohort study that investigates prognosis and risk factors of patients with a TIA, ischemic stroke or intracerebral hemorrhage aged 18 through 49 years. Within this setting we will perform a case-crossover design on potential acute trigger factors. The Medical Review Ethics Committee region Arnhem-Nijmegen approved the study (NL41531.091.12) and all participants will be requested to sign an informed consent form.

### Objectives

The main objective of our study is to determine the risk of mortality and recurrent vascular events in patients with a first-ever young TIA, ischemic stroke or intracerebral hemorrhage.

Secondary objectives are to determine the risk of post-stroke epilepsy, cognitive impairment and (vascular) dementia after a young stroke. The influence of a young stroke on functional outcome, quality of life and depressive symptoms will also be investigated. We will investigate the prevalence of traditional vascular risk factors and the relation between potential acute trigger factors and the occurrence of a young stroke. In addition we will identify baseline characteristics that are associated with our primary and secondary outcomes.

### Study population

#### Patients

All consecutive patients with an acute first-ever young stroke or TIA admitted to the stroke unit or outpatient department of one of the participating centers will be asked to participate in the study. Participating study centers are the Departments of Neurology at the Haga Hospital Den Haag, St Franciscus Gasthuis Rotterdam, Tweesteden Hospital and St. Elisabeth Hospital Tilburg, Catharina Hospital Eindhoven, Amphia Hospital Breda, Medisch Spectrum Twente Enschede, Canisius Wilhelmina Hospital Nijmegen, Rijnstate Hospital Arnhem and Radboud University Medical Centre Nijmegen.

The following Inclusion criteria will be applied:

1. First-ever acute stroke (ischemic stroke or intracerebral hemorrhage) or TIA with corresponding lesion and/or evidence of acute arterial occlusion on CT (A)- or MRI/A-scan.

2. Age 18 through 49 years.

3. Onset of symptoms within 14 days prior to inclusion.

TIA is defined as a rapidly evolving focal neurological deficit, without positive phenomena such as twitches, jerks or myoclonus, with no other than vascular cause lasting less than 24 hours. Acute stroke is defined similar, but with symptoms persisting for more than 24 hours. On the basis of neuro-imaging stroke is further divided into intracerebral hemorrhage and ischemic stroke. Hemorrhagic transformation of an ischemic stroke will be classified as an ischemic stroke.

Exclusion criteria

1. History of a TIA or stroke.

2. Traumatic intracerebral hemorrhage.

3. Any subarachnoid hemorrhage.

4. Intracerebral hemorrhage due to a known ruptured aneurysm.

5. Intracerebral hemorrhage in a known cerebral malignancy (either primary brain tumor or metastasis).

6. Transient monocular blindness or retinal infarction.

7. Cerebral venous sinus thrombosis.

8. Not permanently living in the Netherlands.

#### Controls

Controls will be recruited among patients’ spouses, relatives or social environment. Controls have to be aged 18 through 49 years and will be matched for mean age, sex and level of education. A history of TIA, ischemic stroke or intracerebral hemorrhage is an exclusion criterion, which will be confirmed by a validated questionnaire for verifying stroke-free status [15].

### Procedures

**Figure 1:**
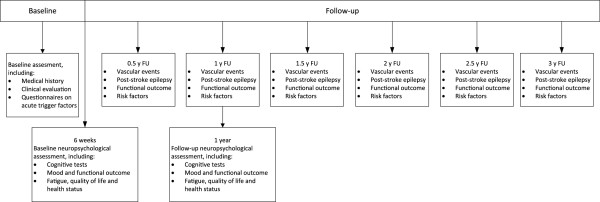
Study design.

All eligible patients will be recruited during the evaluation of their TIA or stroke. Patient recruitment is planned over a 3-to-4 year period and we intend to include 1500 patients. After informed consent and written approval of the participants patients will formally enter the study. If the patient is unable to provide informed consent, consent is provided by the patient’s legally acceptable representative. During admission or visit to the outpatient department, patients will undergo a baseline assessment. In addition, patients will undergo an extensive baseline neuropsychological investigation 6–8 weeks after the index event. The same neuropsychological assessment will be administered 1 year after the TIA or stroke. After baseline assessment, patients will be followed every 6 months for at least 3 years by telephone interview. See Figure 1 for the flowchart of the study design. In addition we intend to include 250 controls who will undergo a baseline assessment on demographics and a neuropsychological assessment.

### Sample size and power calculation

Sample size is based on the least frequent endpoint after young stroke. According to previous literature cumulative incidence of post-stroke dementia is approximately 1% per year. To adjust for the most important confounders (age, sex, stroke severity, education and depression) in our cox proportional hazard analysis, at least 40 cases are needed. To detect a cumulative incidence of 1% per year and a follow-up of 3 years with a power of 80%, results in a sample size of at least 1500 patients.

Control subjects are included to compare cognitive tasks between patients and healthy subjects. Our pilot studies showed that ‘attention’ is the cognitive task which differs the least between patients and healthy subjects (impaired in 14% versus 8% respectively). To identify this difference with a power of 80% and alpha 0.05 in previous mentioned 1500 patients, 265 healthy control subjects need to be included.

### Measures-baseline

#### Medical history

At baseline all participants (patients and controls) will undergo standardized structured questionnaires on demographics, level of education (scored using seven categories in accordance with the Dutch educational system: 1 = less than primary school; 7 = university degree) [16], marital status and employment.

The presence of cardiovascular risk factors will be assessed by standardized, structured questionnaires and classified according to the current guidelines of the American Heart Association [17]. Non-modifiable risk factors include age, sex and a family history of cardiovascular diseases in the next of kin. Well-documented and potentially modifiable risk factors will include cardiovascular diseases (valvular heart diseases, myocardial infarction, coronary artery bypass grafting (CABG), percutaneous transluminal coronary angioplasty (PTCA) and peripheral revascularisation procedures). Furthermore well-documented potential modifiable risk factors will included diabetes mellitus, hypertension, dyslipidemia, smoking and atrial fibrillation.

Less-well documented, potentially modifiable risk factors included a history of migraine, acute infections, the use of oral contraceptives, alcohol consumption and the use of recreational drugs. Patients will be screened for migraine with the MISS questionnaire, a screening instrument regularly used in Dutch hospitals containing questions about migraine in terms of diagnosis, frequency, aura and concomitant symptoms. Definitions of both well-documented and less-well documented, potentially modifiable risk factors are shown in Additional file 1.

In addition to the assessment of risk factors, patients will be asked about a history of epilepsy, pregnancies and complications, pulmonary embolism, deep vein thrombosis and current medication use. Furthermore functional performance prior to the index event will be assessed by modified Rankin scale.

#### Potential trigger factors

In addition to the questionnaires on cardiovascular risk factors, patients will be requested to fill out a standardized structured self-reported questionnaire on acute factors that might trigger stroke. For each trigger factor, patients will be asked to report their usual exposure during the past year and the exposure during a predefined period before the onset of stroke; the hazard period. Hazard periods are based on the estimated duration of the effect of each potential trigger factor as used in previous studies investigating trigger factors of cardiovascular events [13,14,18]. In addition to exposure in the hazard period, patients will be asked about the last exposure before stroke. In this case-crossover design, each patient will serve as his or her own control [19].

Potential trigger factors that will be investigated include smoking, consumption of alcohol, recreational drugs (cocaine, heroin, methadone, amphetamine, cannabis, XTC, anabolic steroids, hallucinogens and EPO), caffeine containing drinks (coffee and cola), valsalva maneuver, fever, positive and negative emotions, physical exercise and sexual activity. Table 1 shows the hazard periods for the different potential trigger factors. To investigate the effect of valsalva maneuver as stroke trigger, patients will be asked about sneezing, coughing, nose-blowing and heavy lifting. The exposure to positive and negative emotions will be measured using the PANAS scale, a 5 level scale which contains 9 positive and 9 negative affects [20]. Whenever patients report a mean score of ≥3.5 on the negative affects, they will be considered exposed to negative emotions [21]. Anger will be measured using the anger scale, consisting of 7 levels of anger [22]. Patients are considered exposed to anger if they report a peak level of anger ≥4 (very angry, furious or enraged). Physical exercise will be expressed in the metabolic equivalent value (MET) according to accepted standards [23]. The exposure of vigorous to extreme exercise (MET ≥ 6) will be reported [18]. Consumption of alcohol, recreational drugs, coffee, cola and smoking will be reported in units per day.

**Table 1:** Hazard period for potential trigger factors

	**Hazard time**
Smoking	1 hour
Alcohol consumption	24 hours
Recreational drugs	4 hours
Consumption of coffee	1 hour
Consumption of cola	1 hour
Valsalva maneuver	1 hour
Fever	24 hours
Positive and negative emotions	1 hour
Anger	1 hour
Physical exercise	1 hour
Sexual activity	2 hours

### Clinical evaluations

#### Physical examination and additional investigations

Baseline standard clinical evaluation will be performed during admission or visit to the outpatient departments. Clinical signs, symptoms and duration of stroke and TIA will be reported. Stroke severity will be assessed by the National Institutes of Health Stroke Scale (NIHSS) [24] and modified Rankin scale (mRS) [25], both measured at admission and at discharge.

Furthermore standard clinical evaluation will include a physical examination and laboratory measures. Additional file 2: Table S1 shows an overview of all investigations. Additional investigational DNA will be stored for future genetic analysis. Patients must consent for storage of the DNA and future analysis.

#### Classification of TIA or stroke: etiology and neuro-imaging

Etiology of ischemic stroke and TIA will be classified according to the TOAST criteria [26], Causative Classification System of ischemic stroke (CCS) [27] and ASCO [28]. Etiology of intracerebral hemorrhage will be classified as hypertensive (deep or infratentorial hemorrhage in combination with hypertension), arteriovenous malformation, cavernous angioma, coagulopathy (iatrogenic or bleeding disorder), central nervous system infection, septic embolism, vasculitis, substance abuse or unknown (cryptogenic, multiple causes and incomplete evaluation) [29]. Etiology will be based on neuro-imaging, medical history and the use of medication.

All patients will undergo neuro-imaging and additionally CT-angiography, MR-angiography or ultrasound will be performed according to standard clinical care. CT- and MRI-scans will be reviewed centrally in the Radboud University Medical Centre. TIA and ischemic strokes will be classified according to arterial territory and addition will be classified as lacunar or territorial. Whenever hemorrhagic transformation of an ischemic stroke has occurred this will be documented.

Intracerebral hemorrhage will be classified as infratentorial (brainstem or cerebellar) or supratentorial hemorrhage (lobar, deep or ventricular) with or without ventricular involvement. Lobar hemorrhage will be further subdivided into frontal, temporal, parietal or occipital. In addition hematoma volume will be calculated according to the A*B*C/2 method [30].

#### Course of the disease

Furthermore the course of the disease during admission will be reported, including medication use, (intra-venous or arterial) thrombolysis or other treatments and complications. Whenever a patient develops a recurrent vascular event (ischemic stroke, intracerebral hemorrhage, TIA, myocardial infarction) or post-stroke epilepsy this will be reported. In addition the course of stroke severity during admission will be assessed by mRS and NIHSS.

### Measures - follow up

All patients will be followed every 6 months by telephone interview. Patients will undergo standardized structured questionnaires on the occurrence of post-stroke epilepsy and recurrent vascular events (TIA, ischemic stroke, intracerebral hemorrhage, myocardial infarction, CABG, PTCA and other revascularization procedures). In addition they will be asked about well-documented potentially modifiable vascular risk factors (diabetes mellitus, hypertension, dyslipidemia and smoking), the use of medication and pregnancies. In case a patient has died, this information will be retrieved from the general practitioner.

As measure of functional outcome mRS and Barthel Index [31] will be administered and patients will be asked about their occupation or education. Occupation will be categorized into 4 skills levels (ranging from first = primary education only to fourth = tertiary education with university degree or equivalent), according to the ISCO-88 (International Standard Classification of Occupations) [32].

### Neuropsychological investigation

An extensive neuropsychological investigation will be administered in all patients six weeks and 1 year after the index event (using parallel versions for some tests that are susceptible to test-retest effects). Controls will be assessed only once at baseline.

The cognitive assessment includes tests used in other large scale epidemiologic studies covering the main cognitive domains [33,34]. Table 2 shows an overview of all cognitive tests performed. Global cognitive functioning will be assessed by the Mini Mental State Examination [35]. Episodic memory will be measured using the 3-trial version of the Rey Auditory Verbal Learning Test [36], which includes a delayed recall and a delayed recognition trial, assessing the acquisition and retention of new verbal information. To assess speed of information processing, Parts I and II of the Stroop Color Word test [37] and the Letter-Digit Substitution Task (an adaptation of the Digit-Symbol Substitution Test [38]) will be used. Visuoconstructive ability will be assessed by the copy trial of the Rey Complex Figure Test [39]. With respect to executive functioning, verbal fluency (animal naming, 60 sec) will be used to test response regeneration, the Brixton Spatial Addition Task [40] will be administered as a measure of concept shifting and rule detection, and the Stroop Interference Score [32] will be included as a measure of response inhibition. Furthermore participants will complete the Star Cancellation of the Behavioral Inattention Test, a short screening battery to assess the presence of a visual neglect [41]. Participants will be tested for language deficits by means of the short version of the Token Test, validated for the Dutch language [42]. To evaluate attention and working memory we will assess the Digit Span subtest from the Wechsler Adult Intelligence Scale - Fourt Edition [43] and the Paper and Pencil Memory Scanning Task (4 subtasks) [44]. Subjective cognitive complaints will be assessed by the Cognitive Failure Questionnaire [45]. The cognitive assessment will be performed in quiet rooms and administered by trained investigators.

**Table 2:** Neuropsychological assessment

**Domain**	**Test/questionnaire**
Cognitive assessment	
Global cognitive functioning	Mini mental state examination
Episodic memory	Rey auditory verbal learning test
Speed of information processing	Stroop color word test
	Letter-digit substitution task
Visuoconstruction	Rey complex figure test - copy
Executive functioning	Animal fluency test
	Stroop interference score
	Brixton spatial anticipation test
Neglect	Star cancellation
Language deficits	Short token test
Attention and working memory	Digit span test
	Paper and pencil memory scanning task
Subjective cognitive complaints	Cognitive failures questionnaire
Mood and functional outcome	
History of depression	
Anxiety and depression	Mini international neuropsychiatric interview
Fatigue	Checklist on individual strength (CIS20r)
Quality of life	EQ-5D
Health status	Stroke impact scale
	SF-12
Functional outcome	Modified rankin score
	Barthel index
	Instrumental activities of daily living

In case cognitive impairment is suspected on the basis of the cognitive screening, relatives will be interviewed on the influence of cognitive performance on daily functioning by means of the IQCODE [46].

In addition to the cognitive screening, questionnaires on mood and functional outcome will be administered. Participants will be screened for a history of depressive symptoms with a standardized questionnaire used in previous large-scale epidemiological studies [47], in which normal reactions to stressful events or normal grief will be excluded. A history of depression is defined as those episodes that require attention of a medical caregiver, including both minor depression and major depressive syndromes as defined by the Research Diagnostic Criteria [48]. Current anxiety and depression will be evaluated by means of the Mini International Neuropsychiatric Interview [49], which is a short diagnostic structured interview based on the DSM-IV. Functional outcome will be assessed by mRS, Barthel Index and Instrumental Activities of Daily Living [50].

Furthermore participants will fill out validated self-report questionnaires on fatigue by means of the Checklist on Individual Strength (CIS20r) [51] and quality of life by means of the EQ-5D [52]. Health status will be assessed by means of the Stroke Impact Scale [53] and SF-12 [54].

### Outcome

Primary outcome will be all cause mortality. Depending on date and location of death information on cause of death will be available either from the hospital (in-hospital mortality) or the general practitioner. Secondary measures of outcome will be the composite endpoint of any recurrent vascular event. Vascular events will include TIA, fatal or non-fatal stroke (ischemic or hemorrhagic), myocardial infarction, CABG, PTCA and other revascularization procedures, whichever occurs first. Stroke and TIA will be defined similar as the index event. Myocardial infarction will be defined by ischemic symptoms with electrocardiographic, cardiac biomarker, or pathological evidence of infarction according to the universal definition of myocardial infarction [55].

In addition, the occurrence of post-stroke epilepsy and dementia will be noted as secondary outcome. Epilepsy will be classified and defined according to the International League Against Epilepsy, in which patients with a single seizure associated with an enduring condition that could cause epilepsy, meet the criteria of epilepsy [56,57]. Dementia will be defined according to DSM-5. Finally, tertiary outcome measures will include functional outcome, quality of life and mood disorders.

Whenever an outcome event is suspected with the aid of standardized structured questionnaires, information from the treating physician will be retrieved. This information will be verified and adjudicated by two independent experienced neurologists or, in case of a myocardial infarction, by a cardiologist, who will be blinded for the index event.

### Analysis

Cumulative risk of mortality, vascular events and epilepsy will be estimated by Kaplan-Meier survival analysis. In the analysis for epilepsy and vascular events, patients who have died will be censored. Cox proportional hazard analysis will be used to calculate hazard ratios for previous mentioned primary and secondary outcome measures adjusted for the necessary covariates. For the outcome measures vascular events and epilepsy, additional competing risk analysis will be performed in which death will be considered as a competing risk as suggested by Fine and Gray [58,59].

For the analysis containing cognition, raw test scores of each test will be calculated and converted to Z-scores using the mean and standard deviation of the controls. Z-scores of tests assigned to the same cognitive domain will be averaged and used in all subsequent analyses as composite Z-score, or domain score. ANCOVA models will be used to compare means of different variables on each cognitive domain adjusted for the necessary covariates. Linear regression will be used to explore the effect of different variables on each cognitive domain and results will be reported as beta coefficients.

For the analysis including acute trigger factors, a case-crossover design as previous described will be used in which each patient will serve as his or her own control [19]. The ratio of the observed exposure frequency in the hazard period to the expected frequency based on the control period will be used to estimate relative risks.

Hazards ratios, beta coefficients and relative risks will be calculated with their corresponding 95% confidence intervals. Comparisons of continuous variables will be done by Student’s t test or analysis of variance or, in case of skewed distributions which cannot be normalized, corresponding nonparametric tests will be used. Chi-squared test will be used for comparison of categorical variables.

## Discussion

ODYSSEY aims to investigate the prognosis after a first-ever young TIA, ischemic stroke or intracerebral hemorrhage in terms of mortality, recurrent vascular events, post-stroke epilepsy and cognitive impairment. Furthermore we intend to determine the prevalence of vascular risk factors in young stroke patients and to relate potential acute trigger factors to the occurrence of a young stroke.

Strong elements of the study are the multicentre prospective design with multiple follow-up assessments. Due to the multicentre approach we will be able to include a large sample size, covering a vast part of the Netherlands including both academic and regional hospitals. The prospective design allows us to obtain an accurate evaluation of the prognosis of young stroke patients, unlike previous small retrospective studies. Furthermore, most previous studies only included patients with an ischemic stroke while we will include patients with a TIA and intracerebral hemorrhage as well. This allows us to study a large part of the spectrum of stroke in young adults. Since patients will only be eligible for inclusion in our study when they have positive brain imaging corresponding with their neurological deficits, ODYSSEY will have a clearly defined population without misclassification. In retrospective studies often a clinical diagnosis was used for defining stroke, leading to a more heterogeneous study population.

Only few small studies among young stroke patients investigated cognitive performance after a young stroke. To our knowledge we are the first to investigate cognitive performance both at baseline as well as 1 year after follow-up. This enables us to describe the course of cognitive performance and potential further decline after an initial assessment. On top of this we will include healthy matched control subjects which will allow for a comparison of cognitive performance with a healthy group.

In addition, this study is the first investigating potential acute trigger factors preceding a stroke or TIA in a non-selected young population. In studies on subarachnoid hemorrhage and ischemic stroke in the elderly, several acute trigger factors already have been identified [13,14]. Due to the case-crossover design we hope to identify possible acute trigger factors, which may give us more insight in the underlying pathophysiological mechanisms of a young stroke. As some of those trigger factors are potentially modifiable, the assessment of acute trigger factors might lead to new prevention strategies in high risk patients.

The estimation of etiology and prognosis is a first step in informing young stroke patients in terms of prognosis. The estimates of recurrent vascular events risks may be used to design future intervention studies on start and withdrawal of secondary prevention in these young patients, as the current prescription of these drugs is based on extrapolated findings from stroke trials in which young patients have been underrepresented or excluded.

In conclusion, ODYSSEY is designed to prospectively determine prognosis after a young stroke and get more insight in etiology of patients with a TIA, ischemic stroke and intracerebral hemorrhage in patients aged 18 through 49 years old in a large sample size.

## Abbreviations

TIA: Transient ischemic attack; ODYSSEY: Observational dutch young symptomatic stroke study; CABG: Coronary artery bypass grafting; PTCA: Percutaneous transluminal coronary angioplasty; MET: Metabolic equivalent value; NIHSS: National Institutes of Health Stroke Scale; mRS: modified Rankin Score.

## Competing interests

The authors declare that they have no competing interests.

## Authors’ contributions

RA participated in the design and coordination of the study and drafted the manuscript MvA participated in the design and coordination of the study and has been involved in revising the manuscript for important intellectual content NS participated in the design of the study and has been involved in revising the manuscript for important intellectual content PB participated in the design of the study and has been involved in revising the manuscript for important intellectual content GvD participated in the design of the study and has been involved in revising the manuscript for important intellectual content RG participated in the design of the study and has been involved in revising the manuscript for important intellectual content TdH participated in the design of the study and has been involved in revising the manuscript for important intellectual content PdK participated in the design of the study and has been involved in revising the manuscript for important intellectual content KdL participated in the design of the study and has been involved in revising the manuscript for important intellectual content AvN participated in the design of the study and has been involved in revising the manuscript for important intellectual content SV participated in the design of the study and has been involved in revising the manuscript for important intellectual content MvdV participated in the design of the study and has been involved in revising the manuscript for important intellectual content RK participated in the design of the study and has been involved in revising the manuscript for important intellectual content EvD participated in the design of the study and has been involved in revising the manuscript for important intellectual content F-EdL conceived of the study and participated in its design and coordination and has been involved in revising the manuscript for important intellectual content. All authors read and approved the final manuscript.

## Pre-publication history

The pre-publication history for this paper can be accessed here:

http://www.biomedcentral.com/1471-2377/14/55/prepub

## Supplementary Material

Additional file 1 **Definition of well-documented and less-well documented modifiable risk factors.**[55,60-65]. Click here for file

Additional file 2: Table S1 Overview of investigations. Click here for file
