# Supplementary material for: Observational Dutch Young Symptomatic StrokE studY (ODYSSEY): study rationale and protocol of a multicentre prospective cohort study
Source: BMC Neurol. 2014 Mar 22;14:55. doi: 10.1186/1471-2377-14-55 (PMC3998025; doi:10.1186/1471-2377-14-55)
Supplement: Additional file 1 — Definition of well-documented and less-well documented modifiable risk factors.[55,60-65]. [file 1471-2377-14-55-S1.docx]

Additional file 1

Definition of well-documented and less-well documented modifiable risk factors

*Well-documented potentially modifiable risk factors:* Myocardial infarction will be defined by ischemic symptoms with electrocardiographic, cardiac biomarker, or pathological evidence of infarction according to the universal definition of myocardial infarction [[55](#_ENREF_55)]. Smoking will be defined as at least 1 cigarette in the 6 months prior to the event. Overweight will be defined as Body Mass Index=25-29 and obesity as Body Mass Index≥30[[60](#_ENREF_60)]. On the basis of laboratory findings diabetes mellitus will be defined as a random blood glucose level ≥ 200 mg/dL (11.1mmol/L) or 2 consecutive fasting venous plasma glucose levels of ≥ 126 mg/dL (7.0 mmol/L)[[61](#_ENREF_61), [62](#_ENREF_62)] or the use of antidiabetics. Dyslipidemia will be defined by either total cholesterol level ≥5.0 mmol/L or low-density lipoprotein level ≥2.5 mmol/L or high-density lipoprotein level <1.0 mmol/L or the use of lipid-lowering drugs. Hypertension will be defined as a systolic blood pressure ≥140 mmHg or diastolic blood pressure ≥90 (measured at least 24 hours after stroke, with 2 independent measures) [[63](#_ENREF_63), [64](#_ENREF_64)] or the use of antihypertensive drugs.

*Less-well documented potentially modifiable risk factors:* Excess alcohol consumption will be defined as consuming more than 200 g of alcohol per week. Furthermore migraine will be defined according to the international classification of headache disorders [[65](#_ENREF_65)].
