# Supplementary material for: Observational Dutch Young Symptomatic StrokE studY (ODYSSEY): study rationale and protocol of a multicentre prospective cohort study
Source: BMC Neurol. 2014 Mar 22;14:55. doi: 10.1186/1471-2377-14-55 (PMC3998025; doi:10.1186/1471-2377-14-55)
Supplement: Additional file 2: Table S1 — Overview of investigations. [file 1471-2377-14-55-S2.docx]

Additional file 2: Table 1. Overview of investigations

|  |  | | **Standard** |
| --- | --- | --- | --- |
| **Physical examination** | Neurological examination | |  |
|  |  | NIHSS | X |
|  |  | mRS | X |
|  | Blood pressure | | X |
|  | Oxygen saturation | | X |
|  | Heart rate | | X |
|  | Body temperature | | x |
|  | Body Mass Index | | X |
| **Additional investigations** | ECG | | X |
|  | Laboratory measures | | X |
|  |  | Fasting glucose | X |
|  |  | Cholesterol levels | X |
|  |  | Blood count | X |
|  |  | Sedimentation rate | X |
|  |  | DNA storage | X |
|  |  | Other specific laboratory measures on indication (young stroke laboratory measures) |  |
|  | Echocardiography on indication | |  |
| **Imaging** | Neuro-imaging^1^ | | X |
|  |  | CT |  |
|  |  | MRI |  |
|  | Vascular imaging^2^ | | X |
|  |  | Angio-CT |  |
|  |  | Angio-MRI |  |
|  |  | Ultrasound |  |

^1^ All patients will undergo neuro-imaging, either CT or MRI scanning.

^2^ All patients will undergo either angio-CT, angio-MRI or ultrasound according to standard clinical care.
